# Locally weighted transmission/disequilibrium test for genetic association analysis

**DOI:** 10.1186/1471-2156-6-S1-S60

**Published:** 2005-12-30

**Authors:** Li Hsu, Xuesong Yu, Jeanine J Houwing-Duistermaat, Hae-Won Uh, Rachid El Galta, Jeremie JP Lebrec, Hua Tang

**Affiliations:** 1 Modeling and Methods, Biostatistics Program, Fred Hutchinson Cancer Research Center, Seattle, WA 98195, USA; 2 Department of Biostatistics, University of Washington, Seattle, WA 98195, USA; 3 Department of Medical Statistics and Bioinformatics, Leiden University, P.O.Box 9604, 2300RC, Leiden, The Netherlands

## Abstract

The transmission/disequilibrium test statistic has been used for assessing genetic association in affected-parent trios. In the presence of multiple tightly linked marker loci where local dependency may exist, haplotypes are reconstructed statistically to estimate the joint effects of these markers. In this manuscript, we propose an alternative to the haplotype approach by taking a weighted average of multiple loci, where the weight is proportional to the product of (*1*-*2X *recombination fraction) and the linkage disequilibrium between markers. As an illustration, we applied the method to the simulated Aipotu data.

## Background

High-dimensional single-nucleotide polymorphism (SNP) data have become increasingly available due to the advancement of high throughput genotyping technologies. These data enable researchers unprecedented capabilities for localizing regions that may be associated with the disease. An often-used strategy for searching for disease-causing genes is to first perform linkage analyses using genome-wide microsatellite or SNP markers to identify a rough candidate region that may harbor the latent disease susceptible gene. In the second stage, dense SNP markers in this candidate region are genotyped so that the location of the disease gene can be further refined. The advantages of this mapping strategy are that it is cost-effective and avoids an untargeted fishing expedition.

In this paper, we focus on the second stage, where a large number of dense markers are genotyped on the study participants. Note that the markers at this stage have already been shown to be closely linked to the disease loci, in other words, linkage analysis has reached its resolution in locating the genes. One may need to rely on the linkage disequilibrium (LD), which measures the allelic association, for further refinement. The LD between a marker locus and the disease locus is thought to decay at a rate of (1-*θ*)^*N*^, where *N *is the number of generations since the introduction of the disease-causing mutation and *θ *is the genetic distance between the two loci. The transmission/disequilibrium test (TDT) [1] that aims to assess the linkage and LD between a marker locus and disease loci has become popular. The TDT has since been extended to multiple tightly linked markers [2] by constructing haplotypes statistically to account for local dependency in the presence of phase ambiguity.

As an alternative to haplotype-based approaches, we proposed an approach that weights the contribution of multiple SNPs according to their association with the locus of interest. This approach does not require determination of haplotypes. The idea is similar to kernel smoothing in nonparametric regression methods [3], where the kernel function is like a sliding window and markers that fall in the window all contribute to the test statistic but with differential weights. The weight here is determined by the distance and correlation of the markers to the locus of interest.

Kofendrerd Personality Disorder (KPD) is a psychiatric syndrome characterized by an overwhelming concern with the meaning of the patient's inner emotions and world view and at the same time subsuming the emotions of others into the self. Nosology for KPD falls into three different groups: 1) "communally shared emotions" symptoms such as joining/founding cults and fear or discomfort with strangers; 2) behavior-related symptoms such as fascination with automobiles and aversion to walking; 3) anxiety-related symptoms such as morbid anger/fear/terror concerning rain/snow and reluctance to wear clothing appropriate for subjective temperature. All three or combination thereof have been used for diagnosis of KPD. The condition is thought to be genetic in origin, possibly exacerbated by prevailing social conditions.

In this paper we analyzed the data collected from the Aipotu country, a populous semi-tropical, semi-desert country with a high prevalence of KPD. The cases were classified as anyone with "notable clusters" of symptoms from any of the three groups as KPD. The families in this dataset were ascertained when at least two siblings could be classified under any of the diagnostic groups or any combination.

## Methods

Consider *K *case-parent trios in which each individual is genotyped with the same *M *autosomal markers at {*t*_1_, ..., *t*_*M*_}. Using Liang et al.'s [4] notation, denote Φ the disease status of the *k*^th ^offspring for *k *= 1, ..., *K*. Let H(*t*) and h(*t*) be the two alleles at marker locus *t*. For simplicity, we use *h *to denote the rare alleles among the affected offspring. This, however, is neither necessary nor consequential. For the *k*^th ^trio, the transmission status *Y*_*k*_(*t*) for paternal alleles at locus *t *can be described as:



Similarly, one can define the maternal transmission status *X*_*k*_(*t*). Assuming that there is only one disease locus at *t*_0 _in the region framed by these *M *markers, the expectation of the transmission status [4] is



where *d*(*t*, *t*_0_) = Pr{*H*(*t*)|*H*(*t*_0_)} - Pr{*H*(*t*)|*h*(*t*_0_)}, a measure for linkage disequilibrium and *θ *is the recombination fraction. We further assume that there is no imprinting in this dataset, that is, *E *{*X*(*t*)} = *E*{*Y*(*t*)}. Denote *C *= *E*{*Y*(*t*_0_)|Φ = 1}. One can see that the value of *C *is determined by the penetrance function and the allele frequencies of disease locus *t*_0 _[4]. Under the assumptions of initial complete LD, random mating, and constant Pr{*H*(*t*_0_)} over time, *d*(*t*, *t*_0_) can be expressed as [5]. Here *N *is the number of generations since the introduction of a disease-causing mutation at location *t*_0_. The parameters of interest in the mean function *μ*(*t*, *t*_0_) are *C *for penetrance, *N *for the number of generations, and *t*_0 _the location of disease locus. Because *Y*(*t*) and *X*(*t*) are potentially correlated over *M *markers, Liang et al. [4] proposed a generalized estimating equation approach to estimate these parameters. An appealing feature of this approach is that the derived parameter estimates remain valid as long as *μ*(*t*, *t*_0_) is correctly specified. Liang et al. [4] also proposed to test the null hypothesis of no linkage or LD to the region framed by the observed M markers by testing *C *= 0. The test statistic is based on a Wald-type statistic, that is, , requiring a simultaneous estimation of (*t*_0_, *N*, *C*) under the assumption that there is a disease locus in the region. However, this approach has several limitations: 1) *t*_0 _is unidentifiable under the null hypothesis; 2) there is a lack of robustness ifthe assumption of constant Pr{*H*(*t*_0_)} over time is not met; and 3) in testing *C *= 0, one would still need to estimate all parameters.

With this consideration we propose to derive a score test statistic for testing *C *= 0 at locus *t*_0_, that is, *t*_0 _is not a disease locus. Based on Equation 10 in Liang et al. [4], a test statistic can be derived as



where

 and superscript *T *indicates the transpose. Under the independence working assumption among *M *marker loci, the test statistic can be further simplified to



One could insert  for *d*(*t*_*m*_, *t*_0_), but it would require a good estimate of *N *as well as the probability of *h*(*t*_*m*_) conditional on *h*(*t*_0_). Instead of estimating *d*(*t*_*m*_, *t*_0_) under a population genetic model, which is often unverifiable, an empirical estimate can be used to quantify the concordance between the two marker loci in the affected offspring. Devlin and Risch [5] provided a comparison of various measures for estimating the LD. Upon a close examination the weight in Equation (1) essentially determines how close marker locus *t*_*m *_is to locus *t*_0_. In other words, if marker locus *t*_*m *_is closer to locus *t*_0_, it is expected that the transmission status at *t*_*m *_would contribute more information to the test statistic at *t*_0_. It then seems logical that one should estimate directly the concordance of the transmission status at locus *t*_*m *_*and *at locus *t*_0_. Since both *X*(*t*) and *Y*(*t*) take discrete values, a natural measure for concordance is the kappa statistic, which is defined as the ratio of the difference between the probabilities of expected and observed disagreements to the probability of expected disagreement. Here, the disagreement between the two marker loci would be the probability of one marker locus transmitting the rare allele, h, whereas the other marker has transmitted the common allele, *H*. Specifically, let *Z*_*k*_(*t*) take value -1 if *X*_*k*_(*t*)*+Y*_*k*_(*t*) is negative, i.e., either both parents transmitting *h *allele but not *H*, or one parent transmitting *h *allele but not *H *and the other parent is non-informative. Similarly, *Z*_*k*_(*t*) takes value 1 if *X*_*k*_(*t*)*+Y*_*k*_(*t*) is positive. Then one can form a 2 × 2 table for *Z*(*t*_*m*_) and *Z*(*t*_0_) at loci *t*_*m *_and *t*_0 _as follows



Define 



Then 

A nice feature of kappa is that the proportion of agreements is calculated after excluding chance agreement. The value of kappa statistic ranges from -1 (negative complete linkage disequilibrium) to 1 (positive complete linkage disequilibrium). Clearly, each term in the sum of Equation (1) remains unchanged if the allele designation, *H *versus *h*, is switched.

It is easy to generalize the test statistic *T*_2 _in a couple of ways. For example, rather than summing over the total *M *markers in the test statistic, one can also use the markers within a prespecified neighborhood of *t*_0_. In addition, the test statistic *T*_2 _can be extended to accommodate multiple affected siblings. The following statistic describes these extensions:



where *n*_*k *_is the number of affected in the *k*^th ^family and B is a pre-specified neighborhood around marker locus *t*_0_. We name test statistic *T *as the *locally *weighted TDT. The choice of the size of a neighborhood depends on many factors such as the nature of the disease mutation and population under study and the marker density. An examination of inter-marker linkage disequilibrium may help determine the window size. By the central limit theorem, *K*^-1/2^*T *is asymptotically normal with a variance that can be empirically estimated by . To account for the multiple comparisons in the tests, one may combine test statistics of all the markers by taking the maximum and determine its critical values by a simulation-based procedure in that the transmission status for each affected offspring are randomly assigned for a large number of times.

## Results

The data that we analyzed in this paper consisted of all affected offspring and their parents from the first replicate of Aipotu study. There were a total of 100 nuclear families with 283 affected offspring. We had no knowledge of the "answers" at the time when we performed the following analyses.

We performed a single-point linkage analysis using the microsatellite markers genotyped on the affected sibpairs (see the companion paper by Houwing-Duistermaat et al. [6]). The microsatellite markers were on average about 7.5 cM apart. We found that the LOD scores for marker D3S0124 and D3S0127 on chromosome 3 were 4.51 and 3.06, respectively. Both exceeded the cut-off threshold of LOD score 3 for IBD testing. Marker D3S0124 was even beyond 3.6, a critical value suggested by Lander and Kruglyak [7] for genome-wide significance. Based on these results, we subsequently purchased 7 packets of basic SNP markers in this region flanked by microsatellite markers D3S0123 and D3S0127. This covers all available SNP markers for the telomere end of chromosome 3. Excluding the microsatellite markers, there were a total of 134 SNP markers covering about 35 cM in genetic distance.

**Figure 1:**
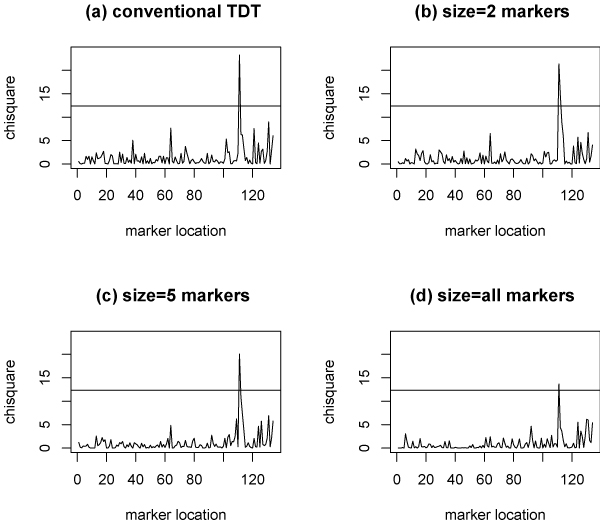
*χ*^2 ^for TDT (left) and smoothed TDT (right).

We applied our proposed test statistics to these 134 SNP markers using all affected-parent trios. We used 2 markers on each side as a pre-determined neighborhood within which the marker contributions to the test statistic are considered. Figure 1 shows the *χ*^2 ^values for the TDT (left panel) and locally weighted TDT (right panel). The lower two plots are the enlarged plots for the 10 markers toward the telomere, some of which showed significant associations with the disease occurrence. The critical values corresponding to level 0.05, indicated by the horizontal lines in the plots, were obtained from the permutation procedure described in "Methods." They were 11.2 and 11.0 for the TDT and locally weighted TDT test statistics, respectively. SNP marker B03T3056 was the only marker that exceeded the threshold for the TDT. Using the locally weighted test statistic both markers B03T3056 and B03T3057 showed significant associations with the disease occurrence in the affected offspring. We have also analyzed the data using larger size windows up to all markers. Although the peak at marker B03T3056 remains significant for all window sizes, the magnitude of the peak decreases with increasing window size (results not shown). Further examination of the pairwise linkage disequilibrium (LD) using HAPLOVIEW (Mark Daly's laboratory, Whitehead Institute for Biomedical Research) indicated an overall very weak inter-marker LD with exception for marker B03T3056 and B03T3057. The LD measure D' between these two markers is 0.60 and the 95% confidence interval is (0.53, 0.67).

To study whether SNP B03T3056 and B03T3057 partly explain the linkage peaks at microsatellite markers D3S0124 and D3S0127, we then included SNP B03T3056 and B03T3057 each and both as covariate(s) in the single-point linkage analysis using the same affected sib pairs as in the initial linkage analysis scan (see Table 1 from Houwing-Duistermaat et al. [6]). The overall LOD score for microsatellite marker D3S0127 and SNP B03T3056 was increased compared to the LOD score for the microsatellite marker only (*p *= 0.02). But the increase in overall LOD score was fairly minimal when SNP B03T3057 was considered. For microsatellite marker D3S0124, only a moderate improvement was observed in the overall LOD scores after including the SNPs. Based on these results, we postulate that SNP B03T3056 only partially explains the linkage signal at microsatellite markers D3S0124 and D3S0127 and other unknown genes may still be present in the region.

## Conclusion

In this paper we proposed a method that accounts for the local dependencies among adjacent markers. We applied it to the simulated dataset and showed that the proposed test statistics yield a smoothed signal between marker B03T3056 and B03T3057. The proposed method did not show much more power than the conventional TDT, in part due to an overall weak inter-marker LD in this SNP dataset (results are not shown). Further work on the performance of the proposed method under a wide range of scenarios will be warranted. The choice of window size in the locally weighted test statistic depends on the nature of the disease mutation and population under study as well as marker density. One possible choice is to first examine an overall LD in the region and use it as guidance for determining the window size. A strong LD suggests a wide window size and vice versa. Another possible choice is to calculate the locally weighted test statistics for a few different window sizes and combine them into one test statistic by taking the maximum. The appropriate critical threshold value needs to be adjusted for such a combinatorial test statistic. In this manuscript, we are testing the null hypothesis *C *= 0. An alternative may be to construct confidence bands for , turning the testing problem into an estimation one. The region for which the confidence bands do not include 0 is likely an indication for a disease locus. An advantage of such a approach is that it provides a confidence interval for which the disease locus might reside. We will investigate methods for constructing confidence bands in the future.

## Abbreviations

SNP: Single-nucleotide polymorphism

TDT: Transmission/disequilibrium test

LD: Linkage disequilibrium

KPD: Kofendrerd Personality Disorder

## Authors' contributions

LH conceived of the study, participated in method development, carried out data analysis, and drafted the manuscript. XY and HT participated in method development. JJH-D participated in method development and data analysis and carried out the joint linkage and association analysis. HWU carried out the linkage disequilibrium analysis. RE acquired the data. JJH-D and JJPL performed the genome-wide scan using microsatellite markers. All authors read, critiqued, and approved the final manuscript.
